# A 3-stage hybrid strategy for heart transplantation following ascending-to-descending aortic bypass grafting

**DOI:** 10.1016/j.jhlto.2026.100486

**Published:** 2026-01-12

**Authors:** Salem T. Argaw, Ali Akamkam, Sébastien Hascoet, Stéphan Haulon, Julien Guihaire

**Affiliations:** a Adult Cardiac Surgery and Transplantation, Marie Lannelongue Hospital, Groupe Hospitalier Paris Saint Joseph, Le Plessis Robinson, France; b Department of Congenital Heart Diseases, M3C Referral Center for Complex Congenical Heart Diseases, Marie Lannelongue Hospital, Groupe Hospitalier Paris Saint Joseph, Le Plessis Robinson, France; c Inserm UMR_S 999, Medical School, University of Paris Saclay, Paris, France; d Vascular Surgery, Aortic Center, Marie Lannelongue Hospital, Groupe Hospitalier Paris Saint Joseph, Le Plessis Robinson, France

**Keywords:** Coarctation of the aorta, Extra-anatomic aortic graft, Ascending-to-descending aortic graft, Hybrid approach, Aortic endovascular repair, Reoperation, Heart transplantation

## Abstract

Cardiac reoperation after an extra-anatomic aortic bypass graft entails considerable risk due to the proximity of the graft to the sternum. In the case discussed here, a woman with 2 prior sternotomies and a retrosternal aortic bypass graft for a history of aortic coarctation presented with advanced dilated cardiomyopathy requiring heart transplantation. The patient was managed through a novel 3-stage hybrid strategy. In the first stage, a catheter-based approach was used to stent the coarcted aorta. Subsequently, the extra-anatomic graft was endovascularly excluded and, finally, the patient underwent reoperation for heart transplantation. The use of interdisciplinary collaboration for coordinated staging and shared decision-making, as in this case, allows for innovative solutions and improved outcomes in complex surgical needs.

## Clinical summary

**Figure 1:**
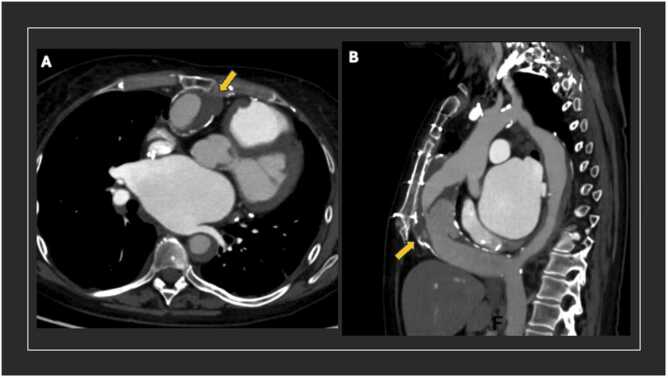
Pre-operative cross-sectional imaging. (A) Axial cut of pre-operative scan demonstrating anteriorly located ascending-to-descending aortic bypass graft (yellow arrow). Semi-circumferential mural thrombus noted as well as atherosclerosis of the graft wall. (B) Sagittal cut of pre-operative scan. Graft proximity to the sternum appreciated. Mural thrombosis of the native descending thoracic aorta also noted.

A 55-year-old woman with a past surgical history of ventricular septal defect closure and reoperation for aortic valve repair as well as extra-anatomic ascending-to-descending aortic bypass graft for coarctation of the aorta (CoA) presented with end-stage heart failure in the setting of dilated cardiomyopathy. After appropriate workup, the patient was listed for heart transplantation; however, preoperative imaging demonstrated that the aortic bypass graft was tightly adherent to the posterior surface of the sternum, posing a high risk for re-sternotomy (Figure 1). Following extensive multidisciplinary discussion, it was decided to proceed with a 3-stage approach to ultimately exclude flow into the graft prior to re-sternotomy.

**Figure 2:**
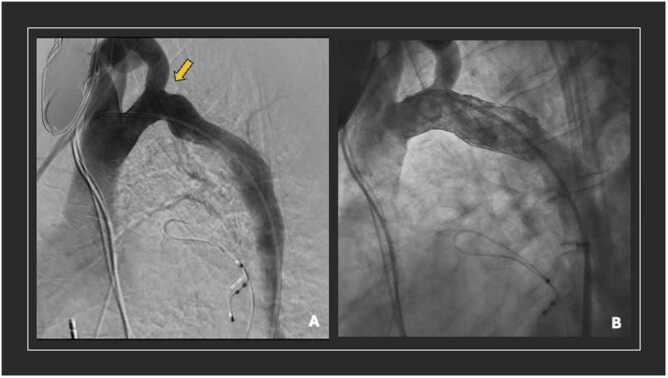
Aortogram pre and post dilatation. (A) Hypoplastic aortic arch demontrasted with coaractation distal to the left subclavian takeoff. (B) Aortogram post intervention shown with improvement in flow through native arch.

**Figure 3:**
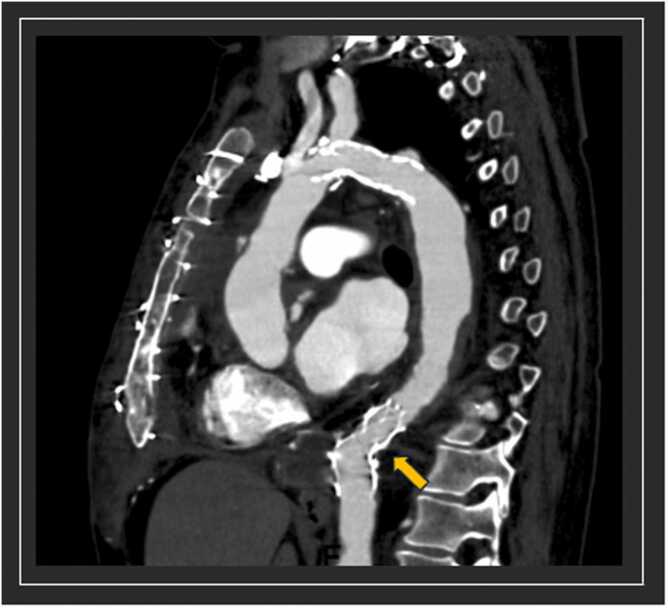
Post-operative cross-sectional imaging. Post transplant scan (sagittal view) demontrasting the stent in the distal arch and the endoprosthesis in the descending aorta (yellow arrow). The non-perfused aortic bypass graft was resected during transplantation.

In the first stage, the patient underwent an aortogram revealing the narrowed arch (Figure 2); however, no pressure gradient was noted (ascending 82/52 (mean 66) mmHg and descending aorta 84/50 (mean 62) mmHg) due to the open extra-anatomic bypass graft. A covered stent, OPTIMUS AT-57XL (JOTEC GmbH, Hechingen, Germany), spanning 22×50 cm was placed just distal to the opening of the left subclavian artery to improve flow in the native descending aorta. There was also no gradient on post-dilation pressure measurement. Recovery post-procedure was appropriate and uncomplicated. Four days later, the patient underwent an endovascular exclusion of the aortic bypass graft. A 38 mm False Lumen Occluder (Candy Plug, Cook Medical, Bloomington, USA) was deployed at the proximal anastomosis to exclude inflow and reinforced with a 20 mm Amplatzer AVP II plug (Abbott, Plymouth, USA). A covered endoprosthesis, ZLBE 24-58 (JOTEC GmbH, Hechingen, Germany), was then deployed in the native descending aorta at the level of the distal anastomosis (Figure 3). Patient required inotropic support with dobutamine following exclusion of the extra-anatomic graft.

Six days after endovascular exclusion of graft, the patient received a donor heart for transplantation. Dense adhesions were present upon sternal re-entry. Cannulation for cardiopulmonary bypass was conducted in the usual manner with the aortic cannula in the ascending aorta and bicaval venous cannulation. Transplant was otherwise uncomplicated. Bypass time was 184 min and cold ischemia time of the donor heart was 207 min. Postoperatively, the patient remained on inotropic support with low dose dobutamine until day 12 in the setting of primary graft dysfunction. The patient was extubated on postoperative day 6 and discharged on postoperative day 23. Patient continues to do well at the 18-month follow-up and provided written informed consent for publication.

## Discussion

Extra-anatomic ascending-to-descending aortic bypass grafting for CoA is a common repair technique with positive long-term outcomes. Recently, Curran et al reported an overall survival following open repair of 94%, 90%, and 85% at 5, 10, and 20 years, respectively.^1^ Nevertheless, the use of catheter-based approach for the management of CoA has steadily risen in the last decade, even becoming the primary approach in adults at many centers. A recent review of outcomes following catheter-based approach (COAST I and II trial) demonstrated durable 5-year relief of obstruction with covered stents but increased late stent fractures, reinterventions, and ∼6% aneurysm formation.^2^ Current AHA/ACC and European guidelines support the use of both approaches; however, the latter favors the catheter-based approach when plausible.^3^, ^4^

In the case described here, a catheter-based approach was utilized for the management of CoA in a patient with complicated surgical history and a retrosternal extra-anatomic aortic bypass graft presenting for heart transplantation. With this 3-stage hybrid approach, the risks associated with tearing the extra-anatomic graft on re-entry were significantly reduced, allowing for a shorter re-entry time and decreased likelihood of low-flow state. Additionally, this approach allowed for a reduced bypass time by avoiding peripheral cannulation and cooling prior to re-entry as well as circulatory arrest. This traditional approach has been associated with bypass times exceeding 290 min and carries the risk of encountering complications such as cerebral malperfusion, kidney injury, and impaired hemostasis.^5^ In a patient with end-stage heart failure, peripheral cannulation also carries the risk of increased systemic afterload and pulmonary edema. Lastly, this approach avoided cross-clamping the extra-anatomic aortic bypass graft and reliance on the native coarcted arch for visceral perfusion, thereby reducing the risk of bowel ischemia.

The 3-stage hybrid approach described in this paper offers an innovative method for approaching reoperation in these high-risk patients. While patients with end-stage heart failure, as in this case, require special consideration, this approach is applicable to similar complex cases requiring reoperation in the setting of an extra-anatomic aortic bypass graft. When available, interdisciplinary collaboration enables coordinated staging and shared decision-making that results in safer procedures and superior patient outcomes.

## Conclusion

This 3-stage hybrid approach for reoperation after ascending-to-descending aortic bypass grafting provides safe and timely re-entry into the chest.
