# Supplementary material for: A 3-stage hybrid strategy for heart transplantation following ascending-to-descending aortic bypass grafting
Source: JHLT Open. 2026 Jan 12;12:100486. doi: 10.1016/j.jhlto.2026.100486 (PMC12890824; doi:10.1016/j.jhlto.2026.100486)
Supplement: Supplementary file 1 — Supplementary material. [file mmc1.pdf]

FORMULAIRE DE CONSENTEMENT POUR LA PUBLICATION D'UNE ETUDE DE CAS

Mme. MYLENE ANDRE,

Vous avez été/êtes prise en charge dans notre établissement, l'Hôpital Marie Lannelongue, pour une transplantation cardiaque. Votre cas présentait une particularité : vous aviez auparavant bénéficié d'un pontage aortique situé en arrière du sternum. Cette configuration chirurgicale inhabituelle a conduit les équipes de l'Hôpital Marie Lannelongue à réaliser une procédure innovante d'exclusion du pontage avant la transplantation cardiaque.

Nous souhaitons partager cette expérience au sein de la communauté scientifique internationale, afin de décrire cette technique et d'en faire bénéficier d'autres équipes confrontées à des situations similaires.

Le Professeur Julien GUIHAIRE, chef du service de Chirurgie Cardiaque Adulte et Transplantation de l'Hôpital Marie Lannelongue vous propose de publier votre cas dans une revue scientifique.

Votre participation à cette étude est **entièrement libre et volontaire**. Vous avez le droit de ne pas participer à cette étude. Dans tous les cas, il n'y aura aucun impact sur votre prise en charge médicale ni sur la qualité des soins et des traitements qui vous seront fournis ou sur votre relation avec votre médecin.

Quel est le but de cette étude de cas?

L'objectif de cette étude de cas est de faire connaître une technique chirurgicale nouvelle, afin d'améliorer la prise en charge de patients présentant des cas complexes comparables au vôtre.

La diffusion de ces résultats au sein de la communauté scientifique pourrait ainsi contribuer à la prise en charge de patients dans des situations similaires.

Comment se déroule l'étude de cas ?

Votre histoire médicale sera présentée de manière anonymisée.
Aucune donnée permettant de vous identifier directement (nom, prénom, date de naissance, numéro de dossier, etc.) ne sera publiée.

Vous recevez ce formulaire d'information, par voie postale, formulaire dans lequel le médecin investigateur vous propose d'utiliser les données de votre dossier médical pour mettre en œuvre cette étude.

Si vous acceptez, **cette étude ne changera rien à votre prise en charge habituelle et n'impliquera aucun autre examen supplémentaire. Seules les données issues de votre dossier médical, nécessaires à la recherche, seront collectées.**

Quelles sont les données recueillies pour cette étude ?

Les données médicales recueillies dans le cadre de cette étude correspondent à vos données démographiques (âge, sexe, IMC), à votre maladie, aux données des examens réalisés dans le cadre de votre prise en charge ainsi que les données relatives à votre chirurgie. Ces données sont collectées directement à partir de votre dossier médical.

Ces données feront l'objet d'un traitement informatisé, anonymisé et confidentiel. C'est-à-dire qu'aucune information portant votre nom, votre prénom, ne sera fournie à quiconque, à l'exception du médecin responsable de l'étude et le personnel autorisé.

Cette démarche est conforme à la réglementation en vigueur, aux articles 6 et 9 du règlement Européen N° 2016/679 (Règlement Général sur la Protection des Données, RGPD) et la loi n°78-17 du 6 janvier 1978 relative à l'informatique, aux fichiers et aux libertés modifiées ainsi qu'à l'engagement de conformité MR004 auquel les Hôpitaux Paris Saint-Joseph et Marie Lannelongue se sont engagés.

Conformément à la réglementation en vigueur (décret n° 2020-077 du 18 juin 2020), les données seront conservées jusqu'à la réalisation du rapport final de l'étude ou jusqu'à 2 ans après la dernière publication des résultats, puis archivées pendant une durée maximale de 20 ans.

Le responsable du traitement des données est le promoteur, la Fondation Hôpital Saint-Joseph, 185 rue Raymond Losserand, 75014 Paris.

Quels sont vos droits ?

Votre participation à cette étude est **entièrement libre et volontaire**. Vous avez le droit de ne pas participer à cette étude.

Cette étude est soumise au Règlement européen (UE) 2016/679 du 27 avril 2016 relatif à la protection des données personnelles (RGPD) et la loi n° 78-17 du 6 janvier 1978 relative à l'informatique, aux fichiers et aux libertés modifiées. Elle s'inscrit dans la méthodologie de référence MR004 de la Commission Nationale de l'Informatique et des Libertés – CNIL (autorité française de contrôle des données personnelles, site Internet : www.cnil.fr) en mai 2018, méthodologie à laquelle La Fondation Hôpital Saint-Joseph, promoteur de la recherche, s'est engagée à être en conformité avec cette méthodologie relative au traitement de données personnelles dans le cadre des recherches dans le domaine de la santé.

Vous pouvez à tout moment vous opposer aux traitements de vos données dans le cadre de cette étude sans justification conformément à l'article 21 du RGPD. Ceci n'aura pas de conséquence ni sur la qualité des soins et des traitements qui vous seront fournis ni sur votre relation avec votre médecin. Les données recueillies jusqu'à l'expression de votre opposition seront utilisées sauf demande expresse de votre part. En effet, conformément à l'article 17 du RGPD, vous avez le droit de demander l'effacement de vos données déjà collectées dans le cadre de cette étude. Votre opposition et l'accord d'utilisation ou non de vos données précédemment recueillies seront tracés dans votre dossier médical.

Conformément aux dispositions des articles 15 et 16 du règlement (UE) 2016/679 du 27 avril 2016 (Règlement Général sur la protection des données) de la loi n° 78-17 du 6 janvier 1978 relative à l'informatique, aux fichiers et aux libertés, modifiée par la loi n° 2018-493 du 20 juin 2018 (décret n° 2018-687 du 1° août 2018), vous disposez d'un droit d'accès, de rectification, de portabilité, d'effacement de celles-ci ou une limitation de traitement (cf. cnil.fr pour plus d'informations sur vos

droits). Vous disposez également d'un droit d'opposition à la transmission des données couvertes par le secret professionnel susceptibles d'être utilisées dans le cadre de cette étude et d'être traitées. Votre demande doit être adressée au **Délégué à la Protection des Données** de la Fondation Hôpital Saint-Joseph, soit par courrier électronique à l'adresse **dpo@ghpsj.fr**, soit par courrier postal à l'adresse : Hôpital Paris Saint-Joseph, Délégué à la Protection des Données 185 rue Raymond Losserand – 75014 Paris.

Vous disposez également du droit d'introduire une réclamation auprès de la Commission Nationale de l'Informatique et des Libertés – CNIL (autorité française de contrôle des données personnelles, site Internet : www.cnil.fr).

Votre dossier médical restera confidentiel et ne pourra être consulté que sous la responsabilité du médecin s'occupant de votre traitement ainsi que par les autorités de santé et par des personnes dûment mandatées par les Hôpitaux Paris Saint-Joseph et Marie Lannelongue pour la recherche et soumises au secret professionnel.

Le Professeur Julien GUIHAIRE, investigateur principal de cette étude sur votre centre, reste à votre disposition pour répondre à vos questions. Il reste votre interlocuteur privilégié pour cette étude. Vous pourrez le contacter à l'adresse mail suivante : j.guihaire@ghpsj.fr

Ce formulaire est rédigé conformément aux recommandations du COPE (Committee On Publication Ethics), disponible sur www.publicationethics.org, publié le 25 février 2016.

CONSENTEMENT POUR UNE ÉTUDE D'UN CAS

Je soussignée Mme Mylène ANDRE accepte librement et volontairement de participer à l'étude de cas intitulée : **Retrosternal Prosthetic bypass of the thoracic aorta in a heart transplant candidate : A multi-stage Hybrid Challenge.**

J'ai pris connaissance de la note d'information dans son intégralité et le médecin m'a informée oralement des modalités de cette étude.

En participant à cette étude, j'accepte le recueil, le traitement, le partage et la conservation de mes données personnelles dans les conditions prévues dans la notice d'information (Protection de vos données personnelles).

J'accepte que mes données médicales, enregistrées à l'occasion de cette étude puissent faire l'objet d'un traitement informatisé par l'Hôpital Marie Lannelongue ou pour son compte.

J'ai compris que ma participation à cette étude de cas est entièrement volontaire et que je peux retirer mon consentement à tout moment. Toutes mes données personnelles soumises seront alors supprimées. Cependant, une fois le rapport de cas rédigé et publié (contenant des données personnelles sous forme anonymisée), il ne sera plus possible pour moi de le retirer. Ma décision n'entraînera aucune pénalité ou perte de bénéfices auxquels j'ai droit, y compris la qualité des soins que je reçois. Le retrait peut être envoyé au médecin responsable de ma prise en charge (veuillez voir les coordonnées ci-dessus).

J'ai compris que :

- ✓ Des photographies, images médicales, vidéos ou illustrations liées à mon cas puissent être utilisées dans la publication.
- ✓ Des images extraites pourront être accessibles via des moteurs de recherche.
- ✓ Une fois la publication effectuée, le consentement ne pourra pas être révoqué.

J'ai bien compris que je détiens les droits sur mes données à caractère personnel suivants :

- ✓ Un droit d'accès à mes données.
- ✓ Un droit de rectifier mes données qui sont inexactes ou incomplètes.
- ✓ Un droit d'effacement de mes données sauf si cet effacement est susceptible de rendre impossible ou de compromettre gravement la réalisation de la recherche.
- ✓ Un droit d'opposition aux traitements futurs de mes données
- ✓ Un droit à la limitation des traitements selon les dispositions légales.

J'ai compris que participer à cette recherche n'engendrera aucun frais et que je ne recevrai aucune indemnisation.

J'ai été informée que mon consentement ne décharge en rien le l'hôpital Marie Lannelongue et l'équipe médicale de l'ensemble de leurs responsabilités et je conserve tous mes droits garantis par la loi.

Partie à remplir par le médecin investigateur

Nom et Prénom du médecin

Professeur Julien GUIHAIRE

Date 21/10/2025

Signature du médecin :

GROUPE HOSPITALIER PARIS SAINT JOSEPH
SITE HOPITAL MARIE LANNELONGUE
133 avenue de la Résistance
92350 LE PLESSIS ROBINSON
Tél : 01.40.94.28.00
92 0 00068 4

Professeur Julien GUIHAIRE
Chirurgien Cardiaque
10100528065

Partie à remplir par le patient

Nom et Prénom du patient

ANDRÉ MYLÈNE

Date 1 / 11 / 2026

Signature du patient :
